# Cannabidiol as an Adjunct to Botulinum Toxin in Blepharospasm – A Randomized Pilot Study

**DOI:** 10.1167/tvst.12.8.17

**Published:** 2023-08-22

**Authors:** Rona Z. Silkiss, Jayson Koppinger, Timothy Truong, David Gibson, Christopher Tyler

**Affiliations:** 1 Silkiss Eye Surgery, San Francisco, CA, USA; 2 Smith-Kettlewell Eye Research Institute, San Francisco, CA, USA; 3 VA Sierra Nevada Health Care System, Reno, NV, USA; 4 The Department of Ophthalmology, California Pacific Medical Center, San Francisco, California, USA; 5 University of California, San Francisco, San Francisco, CA, USA

**Keywords:** blepharospasm (BPS), dystonia, cannabidiol (CBD)

## Abstract

**Purpose:**

The purpose of this study was to evaluate the safety and efficacy of low dose cannabidiol (CBD; Epidiolex) as adjunctive therapy for idiopathic adult-onset blepharospasm (BPS), as well as develop a novel objective assessment methodology to gauge response.

**Methods:**

Prospective, randomized, double-masked, placebo-controlled crossover design of 6 months duration of 12 patients with BPS undergoing routine maximal botulinum toxin (BTX) therapy and experiencing breakthrough symptoms. Participants received their standard BTX every 3 months and were randomized to group A = CBD daily in cycle 1, followed by placebo in cycle 2 or group B = placebo followed by CBD. Videos recorded at days 0, 45, and 90 of each cycle were analyzed to quantify eyelid kinematics. The Jankovic Rating Scale (JRS) was used to provide a clinical rating.

**Results:**

All 12 patients completed the study without adverse events. CBD decreased median eyelid closure amplitude by 19.1% (−1.66 mm, confidence interval [CI] = −3.19 to −0.14 mm, *P* = 0.03), decreased median eyelid closure duration by 15.8% (−18.35 ms, CI = −29.37 to −7.32 ms, *P* = 0.001), and increased the maximum eyelid closure velocity by 34.8% (−13.26 mm/ms, CI = −20.93 to −5.58 mm/ms, *P* = 0.001). The JRS showed a 0.5 reduction in severity and frequency, which was not statistically significant.

**Conclusions:**

Low dose CBD was safely tolerated and improved several BPS kinematic parameters. The clinical scale suggested a direction of effect but may have been underpowered. Further studies are needed to better quantify the clinical relevance.

**Translational Relevance:**

This work describes a novel assessment methodology and therapeutic approach to bBPS.

## Introduction

Idiopathic adult-onset blepharospasm (BPS), previously or also known as benign essential BPS, is an isolated, focal dystonia that causes an abnormal, episodic closure of the eyelids. The etiology is poorly understood, but studies suggest that loss of central nervous system (CNS) inhibition, abnormal plasticity, as well as atypical somatosensory discrimination all likely play a role.^1^^–^^4^ Prevalence varies depending on the study population and assessment criteria but ranges from 20 to 300 per one million.^5^^–^^9^ First-line therapy for BPS is the periodic injection of botulinum toxin (BTX) into or adjacent to the orbicularis oculi muscle every 3 to 4 months.^9^^,^^10^ Despite the efficacy of this therapy, many patients have chronic residual symptoms that are poorly managed with available systemic medications or surgical procedures.^11^^–^^13^

With legalization in many countries, cannabinoids have gained popularity within the general public and medical community. Currently, the US Food and Drug Administration (FDA) has approved Epidiolex, a purified cannabidiol (CBD), for use in treatment of resistant epilepsy syndromes Lennox-Gastaut and Dravet syndrome. Outside of the FDA approval, there have been numerous clinical trials looking at CBD potential in psychiatric disorders, chronic pain, and functional dyspepsia. Combinations of CBD and tetrahydrocannabinol (THC) have been given FDA-approval for the use of the nabiximols, which are combinations of CBD and tetrahydrocannabinol (THC), have been given FDA-approval for alleviating pain and spasticity in multiple sclerosis.^14^^–^^16^ Dronabinol and nabilone are used for the treatment of chemotherapy-induced nausea and vomiting in patients with cancer.^17^

However, cannabinoid application to BPS treatment has not been extensively studied by medical researchers, with only two published studies being noted. In 2017, Harrison et al.^18^ reported symptomatic improvement in BPS in 75% of patients treated with both THC and CBD in a small retrospective chart review. In 2022, Zloto et al. performed a randomized clinical trial that also noted decreased spasms in patients with BPS when given cannabis oil (of which both THC and CBD were used).^19^

We note that there is a significant difference between CBD and THC, which is a common point of confusion to some patients and providers. CBD has low affinity for, and lack of functional activity at CB1 and CB2 receptors, whereas THC has a higher affinity for these receptors^20^^–^^23^ that leads to the psychoactive effects of THC compared to CBD's non-psychoactive effects. In fact, CBD is capable of antagonizing these receptors in the presence of THC.^24^ Recent studies have supported the idea that CBD binds to a distant, allosteric site on CB1 receptors that is functionally distinct from the site for THC. This reduces the potency and efficacy of THC, although the exact target sites for the negative allosteric modulator is presently unresolved.^25^

Due to the high concentration of CBD receptors in the basal ganglia, as well as the muscle relaxant and anxiolytic effects of CBD,^26^^–^^31^ we hypothesized that patients with BPS and residual symptoms despite maximum botulinum treatment might benefit from the use of CBD, particularly with controlled CBD dosing. To assess this hypothesis quantitatively, we devised a feasibility study using a novel method of objectively measuring and analyzing eyelid kinematics using video recording of the periocular region, in addition to the most widely used clinical rating scale, the Jankovic Rating Scale (JRS).

## Methods

This feasibility study was a prospective, randomized, double-masked, placebo-controlled crossover design of 6 months duration with 12 patients from the private practice of the principal investigator (author R.Z.S.). Institutional Review Board (IRB) Approval was provided by Western IRB, as was an Investigational New Drug (IND) Exemption. Clinical trial registration information is publicly available at clinicaltrials.gov, under the identifier NCT04423341.

### Participants

Potential patients were those with a clinical diagnosis of BPS receiving maximally tolerated, routinely scheduled BTX treatment (see the Table). Inclusion criteria were patients in the private practice of the principal investigator with an International Classification of Disease-10th revision (ICD-10) code of BPS (G24.5) or BPS-oromandibular dystonia (G24.4), at least 18 years of age, undergoing routine maximal BTX therapy, experiencing breakthrough symptoms of spasm, and CBD naïve. Exclusion criteria were patients with a concomitant diagnosis of epilepsy, patients who were not CBD or marijuana naive, patients on concurrent anti-epileptics, and patients who were pregnant or wishing to become pregnant. Contact lens wear was not adjusted for the study. Race/ethnicity and sex of the participants were included to identify the heterogeneity/homogeneity of the participants and generalizability to other populations. These were self-reported characteristics. Informed written consent was obtained for all participants. No compensation or incentives were given. This study adhered to the principles of the Declaration of Helsinki and all research activities were Health Insurance Portability and Accountability Act (HIPAA) compliant.

**Table.** Patient Demographics, Botulinum Dose, and Side Effects

Patient	Age	Sex	Race	Treatment	Botulinum	Side Effects
1	72	F	Black	7 y	50	None
2	53	F	White	2 y	50	None
3	76	M	White	15 y	80	None
4	67	F	White	6 y	60	Increase in number of bowel movements; improvement in baseline chronic constipation.
5	80	F	White	20 y	50	None
6	73	M	White	3 y	70	None
7	71	F	White	7 y	30	None
8	58	F	Black	5 y	60	None
9	56	M	Asian	5 y	50	None
10	85	M	White	6 y	40	None
11	67	F	White	18 y	40	None
12	80	F	White	5 y	80	None

## Experimental Design

Participating patients remained on a 3-month BTX injection cycle and were randomized to group A (adjunctive CBD + Botox in cycle 1 followed by placebo + Botox in cycle 2) or group B (placebo + Botox in cycle 1 followed by adjunctive CBD + Botox in cycle 2). Note that although both received BTX, these treatments are labeled “placebo” and “CBD” below for simplicity. Both participants and study staff, including the treating physician, were blinded as to the allocation. Medication was provided by Greenwich Biosciences, Inc. (Carlsbad, CA) in the form of sublingual CBD at 200 mg/day (100 mg BID) under the brand name of Epidiolex. The Epidiolex solution is a clear, colorless liquid with inactive ingredients, including dehydrated alcohol, sesame seed oil, strawberry flavor, and sucralose.

As an initial feasibility trial, dosing was low to establish safety before proceeding to a higher, potentially more efficacious dose (see Discussion). The placebo vehicle oil was also provided by Greenwich Biosciences in an identical vial with instructions to take the corresponding volume of oil. The recruitment and trial began in August of 2020.

Patients underwent BTX injections placed according to the standard protocol developed by Dr. Alan Scott and others.^32^^–^^35^ Patients were injected in the pretarsal and orbital orbicularis oculi. Each point of injection carried four units of BTX in 0.1 cc of non-preserved saline. The zone of diffusion of the Botox was at least 3 mm in diameter from the infusion site.

The end point of BTX treatment was reached when patients achieved sufficient suppression of spasm or poorly tolerated side effects of BTX, such as weak blink, dry eye, or ptosis. Dosing, which had been established for these long-term patients, was continued without change during the study at the participants’ previously established treatment units (see the Table).

**Figure 1.**
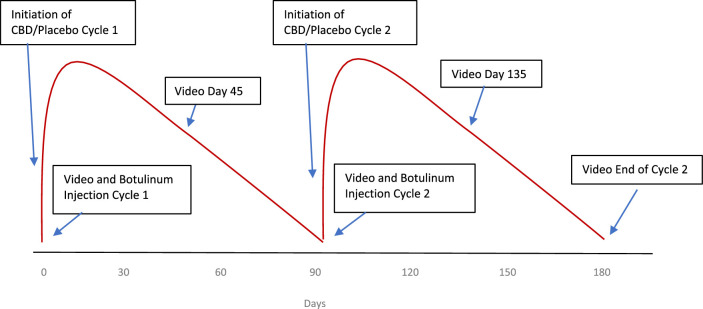
Illustration of the two 3-month cycles to which patients were randomized. The y axis demonstrates the clinical effect of BTX on the orbicularis with the red curves showing the course of effect over the 90 day period.

Patients were treated with BTX at days 0, 90, and 180 and were filmed at the 0, 45-, 90-, 135-, and 180-day time points, as outlined in Figure 1. The y-axis in the figure demonstrates the clinical effect of BTX on the orbicularis, with the red curves showing a rapid increase in effect over the first 1 to 2 weeks, followed by a slow decline over the following weeks, with a minimal clinical effect seen by the 90-day time point.^36^ Patients took daily CBD (100 mg BID) as described above. The primary outcome of safety was evaluated by questioning patients about possible CBD side effects at each session by a medical doctor, as well as self-reporting. The primary efficacy measures were kinematic eyelid analysis of the number of eyelid closures per 100 seconds, eyelid closure duration, eyelid closure amplitude, and maximum eyelid closure velocity, as detailed below. The trial was completed by April of 2021.

## Eyelid Closure Analysis

All patients underwent video recording with the use of a high-resolution commercially available video camera (Panasonic HC-V770; Osaka, Japan) to capture the eyelid positions at a sampling rate of 30 frames per second. Patients were seated at the slit lamp with their head position verified and were assessed in three different lighting conditions – in regular examination room lighting, under examination with the glare source of an indirect ophthalmoscope (at 2000 lux on both eyes from 5 feet), and in dim lighting. They were given separate tasks during each lighting condition in which blink dynamics were assessed – a quiescent state that consisted of focusing on a fixation target, and two conversation states during which the facial features were in substantial motion. These two conversation states were termed “pleasant” and “unpleasant” lines of questioning (see supplementary materials) in an attempt to identify any anxiolytic component.

**Figure 2.**
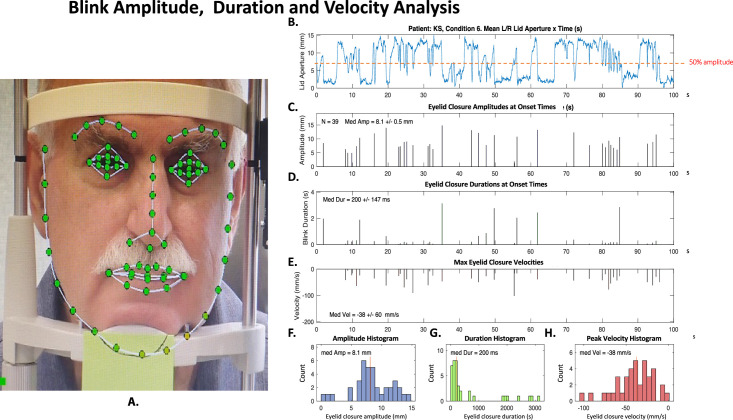
Eyelid closure parameter analysis. (**A**) Single frame application of face template. (**B**) Time series of average palpebral aperture. (**C, D, E**) Estimated eyelid closure amplitudes, durations, and velocities at their occurrence times. (**F, G, H**) Resultant eyelid closure amplitude, duration, and velocity histograms.

The upper and lower eyelid positions captured from each frame of the videos were then input into custom software developed by Visage Technologies (Diskettgatan 11A, SE-583 35; Linköping, Sweden), which fit a feature template to the facial features in each frame, including the upper and lower lids of each eye, as shown in Figure 2A. The difference between the upper and lower lid positions defined the lid aperture, also known as the palpebral fissure. The eyelid closure parameters were then calculated from the eyelid aperture time series with custom software written in MATLAB (MathWorks, Natick, MA).

The derivation of eyelid closure amplitude and duration from the eyelid aperture time course is illustrated in Figures 2B to 2H. The time courses were cropped to the last 3000 frames (100 seconds) of each video recording in order to provide a uniform time base for the eyelid closure frequency analysis. An eyelid closure was defined as less than 50% of the maximum vertical extent of the palpebral aperture during this recording period (see dashed red line in Fig. 2B), with the duration specified as the full width at half-height of the (negative) eyelid closure time course. The eyelid closure amplitude was specified as the minimum aperture at any point during this duration relative to the pre-closure aperture (defined as the maximum aperture in the period between the preceding closure and the current closure).

Examples of the onset times and amplitude estimates for each eyelid closure are plotted as the line graph in Figure 2C, with the closure duration estimates plotted similarly in Figure 2D, and closure velocity estimates in Figure 2E. The resulting histograms of the amplitudes, durations, and velocities of the eyelid closures are plotted in Figures 2F, 2G, and 2H, respectively, with the outcome variable of their medians shown by the vertical red line in each panel.

From these calculations, the number of eyelid closures and the median amplitudes, durations, and velocities in each viewing condition and study phase were then presented for statistical analysis to determine the significant effect of the CBD intervention.

### Clinical Rating Scale

In addition to the computer analysis, the raw video data generated from the recording session as described above was trimmed into a 2-minute clip that involved a fixation task in natural lighting. These 60 separate video clips were then randomized and uploaded to a pair of blinded clinicians for review. The clinicians graded each participant's session with a severity and frequency scale as outlined by the JRS. The scores with corresponding time points were then returned to their original chronological order and underwent statistical analysis as detailed below.

### Statistical Analysis

Analysis of the eyelid closure data from the crossover design was performed using R software, version 4.0.2. The analysis was applied to the parameters derived from video analysis: number of eyelid closures per 100 seconds, median eyelid closure duration, median eyelid closure amplitude, and median max eyelid closure velocity. Linear mixed-effects regression (LMER) models were fitted utilizing the R lme4 package. The residuals met conventional criteria for the regression model assumptions. Additionally, the homogeneity of variance assumption was evaluated using Leven's test and the assumption of normality was assessed with Shapiro-Wilk normality tests. Multiple comparison tests to find significant differences between the treatment group means were performed using the Bonferroni-Holm method and by-passing model estimates through ANOVA. Carryover effects were evaluated by assessing the significance of a treatment-session interaction term and by comparing those who received CBD in session 2 with those who received CBD in session 4 by *t*-test.

To avoid fitting an overly complex model, variables were selected a priori and evaluated for significance. Each variable was evaluated to see how its inclusion affected the model's Akaike information criterion (AIC) score, coefficient of determination (R^2^), and *P* value (the cited *P* values are 2-sided without adjustment for multiple analyses).

**Figure 3.**
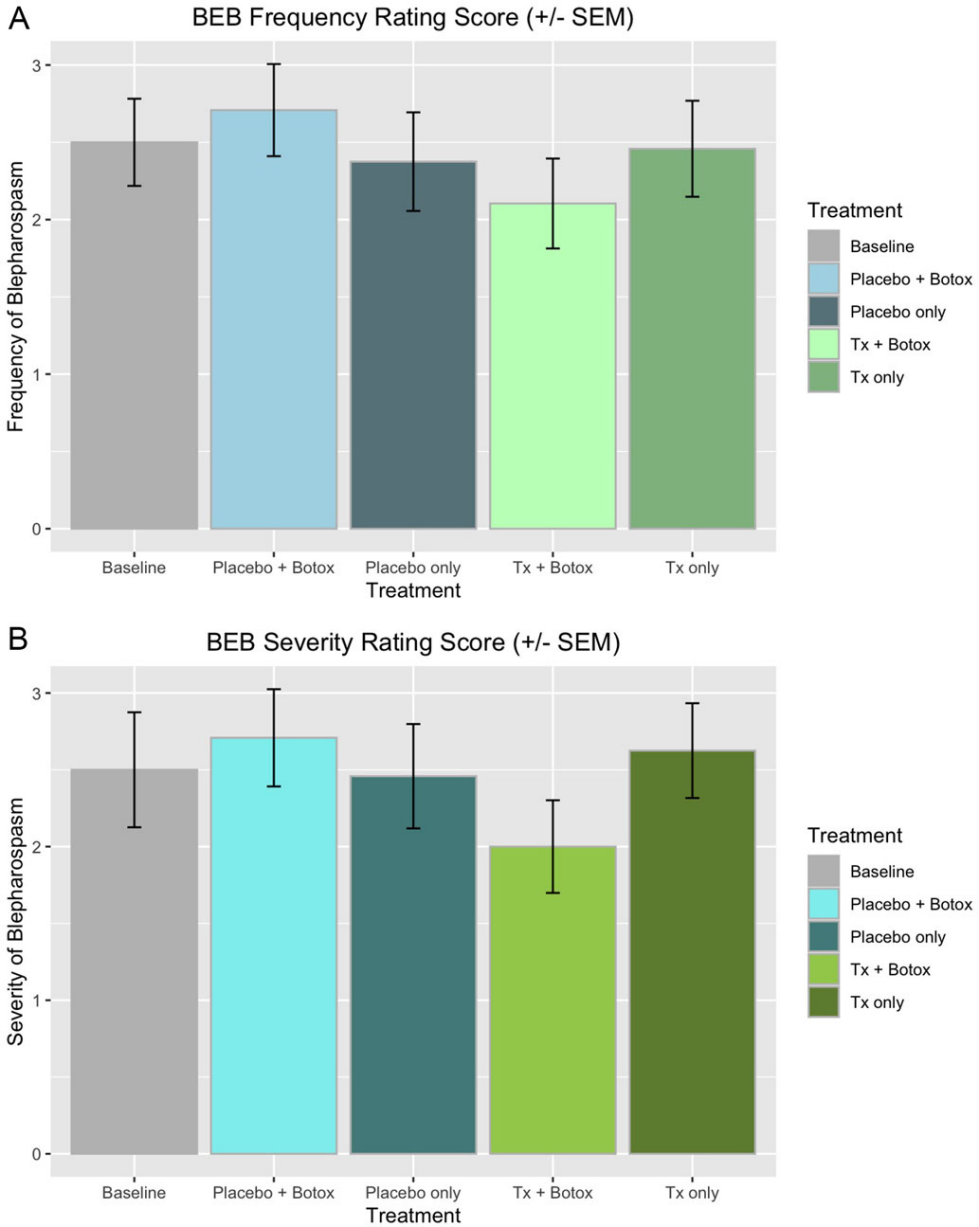
Results of JRS clinical scale. (**A**) Frequency; (**B**) Severity.

To further assess the clinical implications of CBD in patients with BPS and to validate the data generated from our eye tracking software, we performed a subanalysis on the data from the JRS. The JRS data were analyzed by means of ANOVA, LMER, and multiple comparisons of means using Tukey's contrasts. Visual representation of the JRS data and SEM can be found in Figure 3.

## Results

Twelve patients were enrolled in the trial (8 women and 4 men) and all patients completed the trial through the final end point. The median age of patients enrolled in the study was 71.5 years. Median treatment time on maximally tolerated BTX was 6 years (2 – 20 years). All patients tolerated CBD and BTX without any significant adverse events. One patient reported increased well-tolerated stool softness. See the Table for detailed demographic information.

**Figure 4.**
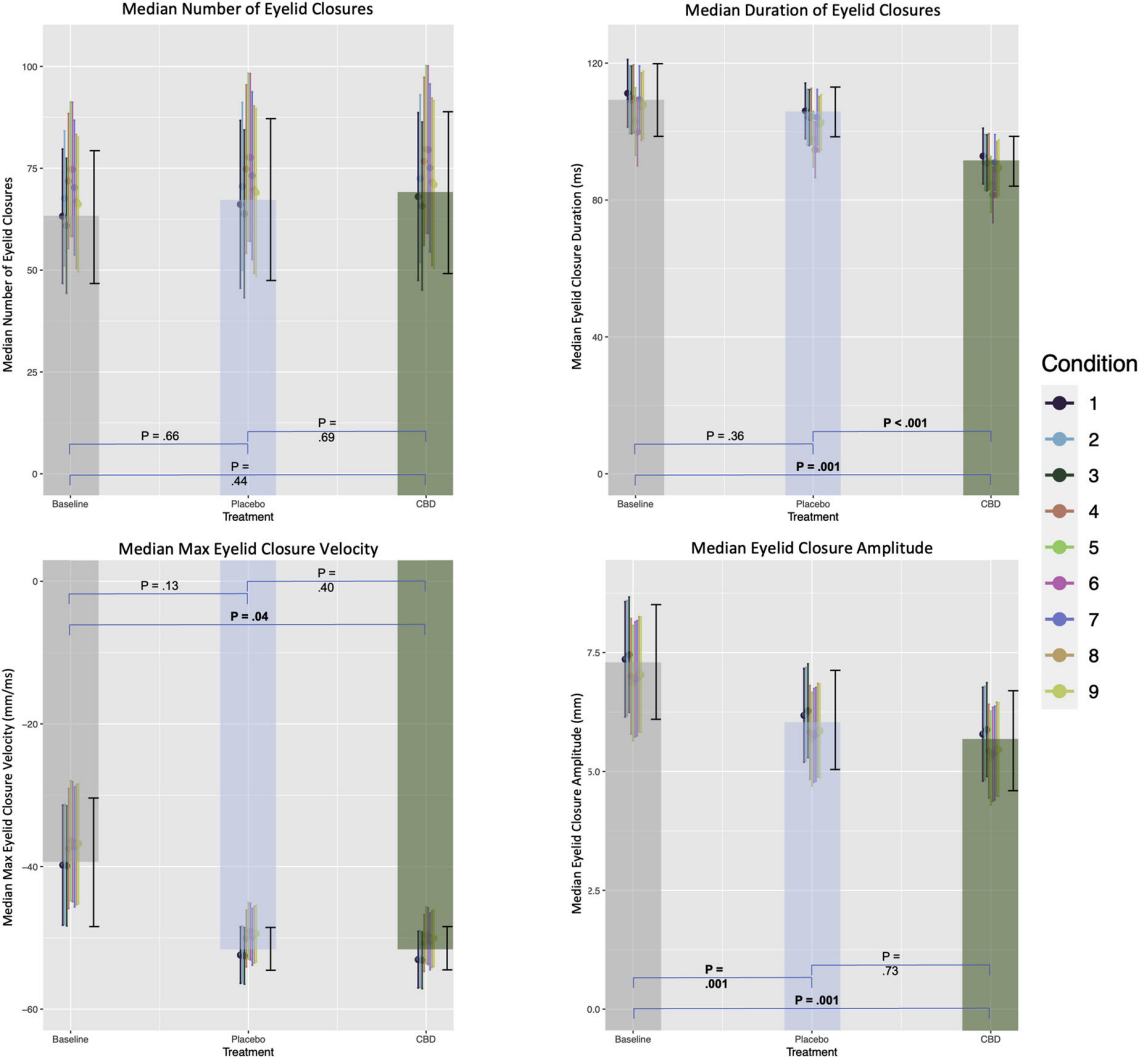
The Y axis conveys the estimated average given the change from baseline measure. Standard error (SE) bars for each condition are in color; SE for each treatment group is in *black*. Contrast analysis between groups is at the base.

The dataset comprised a total of 1,620,000 video frames across the 9 conditions at 5 time points for the 12 participants. The eyelid closure analysis was found to capture 100% of the recorded eyelid position within the analysis range in every lighting and task condition. Summary statistics of the various outcomes calculated from the analysis can be found in the Supplementary Table S1, as well as the plotted LMER estimates in Figure 4. Overall, there were significant differences between median eyelid closure amplitude and median max eyelid closure velocity. There was also a difference between treatment groups for median eyelid closure duration even after using a more stringent, single-step adjustment procedure for multiple analysis. There were no significant differences between treatment groups and baseline for number of closures per 100 ms.

According to the LMER model, the application of CBD decreased median eyelid closure duration by 15.8% (−18.35 ms, confidence interval [CI] = −29.37 to −7.32 ms, *P* = 0.001), whereas the placebo generated no significant change over the baseline score by 4.6% (−5.15 ms, CI = −16.16 to 5.86 ms, *P* = 0.36). Both analyses of variance and multiple comparisons of means showed statistically significant differences between CBD and placebo (*P* = < 0.001), CBD and baseline (*P* = 0.001), but not placebo and baseline (*P* = 0.36). Furthermore, our model estimates that BTX reduced median eyelid closure duration by approximately 6.5% (−7.18 ms, CI = −13.00 to −1.35 ms, *P* = 0.02) and that dim lighting conditions with pleasant and unpleasant questions were also significant for reducing closure duration by 7.5% (−8.25 ms, CI = −14.61 to −1.89 ms, *P* = 0.01) and 10.3% (−11.32 ms, CI = −14.61 to −1.89 ms, *P* < 0.001), respectively.

Our mixed effects regression model estimated that median eyelid closure amplitude was reduced by 19.1% (−1.66 mm, CI = −3.19 to −0.14 mm, *P* = 0.03) when patients received CBD. However, contrast analysis determined that there was no significant difference between CBD and placebo (*P* = 0.08) or CBD and baseline (*P* = 0.07) for this metric.

There were significant reductions from baseline for the median max eyelid closure velocity in both the CBD and placebo groups. Our model estimates that CBD increased the median eyelid closure velocity by 34.8% (−13.26 mm/ms, CI = −20.93 to −5.58 mm/ms, *P* = 0.001), whereas the placebo increased it by 33.2% (−12.62 mm/ms, CI = −20.29 to −4.95 mm/ms, *P* = 0.001). Analysis of variance and multiple comparisons of means tests confirmed that there was a statistical difference between placebo and baseline (*P* = 0.003), CBD and baseline (*P* = 0.002), but not between CBD and placebo (*P* = 0.93). Additionally, all conditions apart from the first three showed significant reductions in median max eyelid closure velocity (see conditions 4–9 in Supplementary Table S1). When adjusting for multiple comparisons via single-step procedure, median duration of eyelid closures was the only kinematic measurement that remained significantly different (<0.05) between CBD and placebo as well as CBD and baseline. The LMER model coefficient of determination explained approximately 90% of the variance for all outcomes apart from the number of eyelid closures recorded.

With respect to the estimated blink parameters, the washout period in this trial design appeared to be sufficient to prevent a carryover effect. The interaction term between treatment and session (time period) was not significant when evaluated in any of the outcomes. Individual *t*-tests between the two treatment orders (CBD|Placebo and Placebo|CBD) were also nonsignificant, indicating that there was no difference between the two arms of the trial.

The statistical analysis of the JRS results was potentially underpowered given the limited number of data points and showed no statistically significant change from baseline. When looking at median values, however, there was, on average, a 0.5 reduction in severity and frequency from baseline in the treatment groups, suggesting a direction of effect (see Fig. 3).

Initially, a large series of published surveys to track patient experience were utilized at the same appointment as their eyelid closure analysis. These were filled out by the participants themselves, with data compilation and analysis at the end of the study. Many patients noted that they found the questions confusing, complained of survey fatigue, and were reluctant to answer the surveys fully. These results were not utilized due to incomplete survey completion. Participants were asked at each visit whether they were experiencing side effects. None were reported outside of one patient noting increased stool softness.

## Discussion

Despite recreational legalization in several states, and growing attention over the last several decades, cannabinoids have been poorly studied in the medical literature. This lack of research can be attributed to the fact that usage of marijuana-derived products still constitutes a federal offense, and because there has been little distinction made between different cannabinoids.^37^^,^^38^ Although both THC and CBD are cannabinoids, only the former has the well-known psychoactive properties.^19^^–^^23^

Additionally, BPS itself is a challenging disease to accurately quantify. The difficulties of clinical assessment have led to numerous clinical rating scales, many of which have reasonable levels of internal consistency, but poor inter-rater agreement.^39^^–^^42^ This provided the rationale for our development of a novel objective method of measuring eyelid kinematics. In our study, high-speed video was successfully able to track and objectively measure kinematic responses in BPS. This approach may serve as an effective method for objective measurement of the response to treatment in future studies.

Daily 200 mg of sublingual CBD was found to be well-tolerated, without adverse events, and provided statistically significant improvements in several BPS parameters – namely median maximum eyelid closure velocity and median duration of eyelid closures. There was either no significant differences or the only difference was between baseline and placebo in number of eyelid closures and median eyelid closure amplitude, suggesting that Botox worked as we would have expected. It is not yet clear if there is a linear relationship between the change in magnitude of these parameters and a clinically significant improvement in patients with BPS. Our clinical rating scale, JRS, hinted at a modest change, but was not statistically significant. This was not unexpected given the underpowered nature of the study to assess treatment effect. The JRS has been argued by some to have a poor definition of spasm, with a lack of attention to specific clinical features, and poor sensitivity to smaller changes in severity and frequency. It was used in this study, as historically, it has been the most widely used severity scale. A more modern scale^43^ may have provided different results, but it is also entirely possible that the change in these kinematic measures is not clinically relevant.

Although our digital video strategy has the potential to measure a significantly higher number of variables present during the motion of the eyeblink, our small team and scope were limited in this initial study. Nevertheless, our dataset included 1.6 million video frames from 12 patients, and running quantitative analyses is time-intensive. With scant support from the literature regarding kinematic analysis, we constructed a short list of applicable variables and analyzed the four reported. We suspect this type of analysis will grow more popular, and a recent article has used similar variables.^44^

Whereas the increased eyelid closure velocity and decreased duration are more easily interpreted in the context of BPS, the change in amplitude of the closure is more challenging. One might assume that spasms tend to decrease the amplitude, but we noted consistent high amplitude closures in some patients, and preceding spasms in others. Some data has suggested that hyper-excitability of brainstem neurons in BPS can lead to an increase in the amplitude of the closure reflex and a prolongation of the duration of the eyelid closure.^45^

Additionally, we included conversation states and lighting conditions as covariates in our analysis to account for their potential influence on the outcomes of interest. Upon examining the coefficients and *P* values associated with these covariates, we observed that conditions 2 and 3 (unpleasant/pleasant conversations at room lighting) did not show a significant effect. However, for all kinematic metrics, both conditions 5 and 6 (unpleasant/pleasant conversations at dim lighting) exhibited a significant effect. Additionally, for median eyelid closure velocity, we observed a significant effect with unpleasant questions in both conditions 8 and 9 (unpleasant/pleasant conversations at bright lighting). The only instance where the estimated coefficient for a conversation condition was significant for unpleasant questions and not for pleasant questions was in median eyelid closure amplitude. It is noteworthy that the coefficients for positive and negative questions were generally very close in magnitude, even in the case of median eyelid closure amplitude, suggesting a lack of or limited effect attributable to the conversation state, but rather an effect influenced by the lighting source. This is not surprising, as photophobia is a commonly reported symptom in BPS, and several studies have suggested that bright lights may even precipitate spasm.^46^^–^^48^ A prior case-control study has suggested that the wavelength of light exposure may be important and could be treated with lens tint.^49^ Our results suggest decreasing the intensity of the light would be beneficial as well.

The dosage in this study was limited by safety considerations, as this was an initial feasibility study. The authors recommend a future study of up to five times the current study's dosing (which more closely approximates the dosing used in clinical practice for epilepsy)^50^^,^^51^ to determine if this would provide further symptom reduction without additional significant side effects.

Of note, blink time courses in patients with BPS can be highly idiosyncratic, with extended durations from partially closed lids, and multiple lid fluttering behavior that does not conform to the concept of discrete closures. Under these circumstances, no definition of a discrete “eyelid closure” can be expected to be fully satisfactory under all circumstances. The 50% amplitude criterion used and discussed in Methods was found to perform well for all participants. The example in Figure 2B illustrates the combination of short closures, extended eye closures, and fluttering that can occur in these patients. It should be noted that, of the “closures” identified according to this procedure, shorter measurements were thought to be representative of regular closures whereas longer measurements may be considered as either episodes of short or long BPSs. Further study with subgroup analyses of the variety of types of spasms is likely to be helpful in the future.

Regarding the 30 Hz sampling rate of the analysis, although not ideal for specifying fine details of regular eyelid closures time courses lasting 100 to 200 milliseconds, the rate is sufficient to ensure that all closures were captured, and to specify the durations of eye closures longer than typical to a sampling resolution of 33 ms. Given this, we found that the high-resolution video recording analysis was able to track and measure kinematic responses in BPS, and may serve as an effective method for objective measurement of the response to treatment in future studies.

There are many alternatives for the time frames and testing periods of our study. Notably, in the clinical trials of Epidiolex for epilepsy, the maintenance period was 12 weeks and changes in epileptic activity were noted to occur within 4 weeks.^50^^,^^51^ Although a longer trial period of at least 6 to 12 months in our study would better assess variability in BPS and the BTX effects, the present results validate our expectation that testing rounds of 1 to 2 months were sufficient for a feasibility study. No BTX washout period was used in an attempt to keep patients’ normal BTX injections on the standard schedule. This choice was validated by the absence of a significant difference between groups A and B prior to the onset of each arm of the design. Washout of CBD prior to the subsequent recording evaluation was deemed sufficient based on Epidiolex's reported half-life of 56 to 61 hours.^52^

Epidiolex CBD dosing was selected based on internal data from our original, retrospective review^53^ which demonstrated subjective improvement in BPS symptoms in patients treated with CBD in the 100 to 300 mg range, without notable side effects. Although this dose was lower than Epidiolex dosing in pivotal epilepsy trials,^50^^,^^51^ it served as a good benchmark for the safety and tolerability of the drug in a non-convulsive setting.

The small sample size itself was deemed appropriate for a feasibility study. It was practical for an early-stage investigation within a single clinical location, whereas providing the critical preliminary information for planning subsequent larger studies.

Finally, although the authors understand that clinical relevance is of particular importance, we believe that BPS is a complex condition in which the traditional “gold standard” of clinical assessment is inadequate. Most clinical assessment scales are subjective and have difficulty interpreting the wide and varied movements that we see in BPS. We believe our objective kinematic analysis is a more sensitive and precise method of quantification, and with further time and study may provide the basis for a more accurate assessment.

In summary, despite the study limitations, this pilot feasibility study demonstrated via a unique kinematic objective analysis that low dose CBD may be a useful adjunct to traditional BTX injections for the treatment and symptom abatement of idiopathic adult-onset BPS. Further study with higher dosing is warranted.

## Supplementary Material

Supplement 1
