# Supplementary material for: Cannabidiol as an Adjunct to Botulinum Toxin in Blepharospasm – A Randomized Pilot Study
Source: Transl Vis Sci Technol. 2023 Aug 22;12(8):17. doi: 10.1167/tvst.12.8.17 (PMC10461691; doi:10.1167/tvst.12.8.17)
Supplement: Supplement 1 [file tvst-12-8-17_s001.pdf]

Supplemental Material

	Number of Eyelid Closures				Median Duration of Eyelid Closures				Median Eyelid Closure Amplitude				Median Maximum Eyelid Closure Velocity			
**Linear Mixed Effects Regression**																
**Predictors**	**Estimates**	**SE**	**CI**	**P**	**Estimates**	**SE**	**CI**	**P**	**Estimates**	**SE**	**CI**	**P**	**Estimates**	**SE**	**CI**	**P**
Intercept	119.93	51.15	19.69 – 220.17	.02	110.75	20.47	70.63 – 150.87	.001	8.71	1.74	5.29 – 12.14	.001	-66.56	12.14	-90.41 – -42.71	.001
Placebo	2.7	6.43	-9.91 – 15.31	.44	-5.15	5.62	-16.18 – 5.86	.36	-1.38	0.78	-2.88 – 0.17	.08	-12.62	3.91	-20.29 – -4.95	.001
CBD	4.99	6.43	-7.61 – 17.60	.66	-18.35	5.63	-29.37 – -7.32	.001	-1.66	0.78	-3.19 – -0.14	.03	-13.26	3.91	-20.93 – -5.58	.001
Age	-0.81	0.72	-2.23 – 0.61	.26	-0.08	0.28	-0.64 – 0.48	.79	-0.01	0.02	-0.06 – 0.03	.56	0.38	0.16	0.06 – 0.70	.02
Botox	-4.03	3.08	-10.07 – 2.01	.19	-7.18	2.97	-13.00 – -1.35	.02	-1.93	0.66	-3.22 – -0.64	.003	3.28	1.33	0.66 – 5.90	.01
Condition 2	4.42	4.67	-4.74 – 13.58	.34	-1.85	3.26	-8.24 – 4.54	.57	-0.31	0.37	-1.03 – 0.42	.40	0.1	1.3	-2.46 – 2.65	.94
Condition 3	-2.31	4.71	-11.55 – 6.93	.62	-1.94	3.26	-8.33 – 4.45	.55	-0.21	0.37	-0.93 – 0.51	.57	-0.1	1.3	-2.65 – 2.45	.94
Condition 4	8.66	4.72	-0.59 – 17.90	.07	-1.58	3.26	-7.97 – 4.81	.63	-1.66	0.37	-2.38 – -0.93	.001	2.32	1.3	-0.24 – 4.87	.08
Condition 5	11.57	4.67	2.41 – 20.73	.01	-8.25	3.25	-14.61 – -1.89	.01	-1.27	0.36	-1.97 – -0.56	.001	3.43	1.29	0.89 – 5.97	.008
Condition 6	11.49	4.67	2.33 – 20.65	.01	-11.32	3.25	-17.68 – -4.96	.00	-1.09	0.36	-1.79 – -0.38	.003	3.29	1.29	0.75 – 5.83	.01
Condition 7	7.06	4.69	-2.14 – 16.25	.13	-1.85	3.25	-8.21 – 4.51	.57	-0.42	0.36	-1.13 – 0.28	.24	2.55	1.29	0.01 – 5.09	.05
Condition 8	3.6	4.65	-5.51 – 12.72	.44	-3.92	3.25	-10.28 – 2.44	.23	-0.72	0.36	-1.43 – -0.02	.04	2.86	1.29	0.32 – 5.40	.03
Condition 9	2.93	4.69	-6.27 – 12.12	.53	-3.37	3.25	-9.73 – 2.99	.30	-0.42	0.36	-1.13 – 0.28	.24	3	1.29	0.46 – 5.54	.02
ICC	.46				.91				.88				.85			
$N_{Patient}$	12				12				12				12			
Observations	537				537				537				537			
Conditional R^2	0.6				0.92				0.9				0.86			
**Multiple Comparison Tests**																
**Contrasts**	**Estimates**	**SE**	**Z-Score**	**P**	**Estimates**	**SE**	**Z-Score**	**P**	**Estimates**	**SE**	**Z-Score**	**P**	**Estimates**	**SE**	**Z-Score**	**P**
Baseline - Placebo	2.92	6.71	0.44	.90	-5.15	5.62	-0.92	.61	-1.38	0.78	-1.75	.16	-12.61	3.90	-3.23	.003
CBD - Baseline	4.84	6.71	0.72	.76	-18.35	5.63	-3.26	.003	-1.66	0.78	-2.14	.07	-13.25	3.91	-3.39	.002
CBD - Placebo	1.92	5.92	0.32	.94	-13.20	2.22	-5.95	.000	-0.31	0.15	-2.08	.08	-0.64	1.85	-0.35	.93
**Anova**																
**Predictors**	**Chisq**	**Df**	**P**		**Chisq**	**Df**	**P**		**Chisq**	**Df**	**P**		**Chisq**	**Df**	**P**	
CBD	0.60	1	.44		7.82	1	.005		5.53	1	.02		11.53	1	.001	
Placebo	0.18	1	.67		0.15	1	.70		3.49	1	.06		10.45	1	.001	
Botox	1.71	1	.19		6.47	1	.01		8.28	1	.004		6.04	1	.01	
Condition	16.86	8	.03		20.39	8	.009		30.17	8	.000		21.12	8	.007	
Age	1.25	1	.26		0.28	1	.59		1.85	1	.17		5.56	1	.02	

Table 2: Statistical analysis comprised of linear mixed effects model regression (lmer) estimates, multiple comparison contrast tests between treatment groups, and analysis of variance utilizing type II sum of squares.

Post hoc, multiple comparison analysis P values have been adjusted via single-step method.
ICC - Intraclass Correlation Coefficient
Condition 1 - Room lighting, fixation target
Condition 2 - Room lighting, unpleasant questions
Condition 3- Room lighting, pleasant questions
Condition 4- Dim lighting, fixation target
Condition 5 - Dim lighting, unpleasant questions
Condition 6 - Dim lighting, pleasant questions
Condition 7 - bright lighting, fixation target
Condition 8 - bright lighting, unpleasant questions
Condition 9 - bright lighting, pleasant questions

eTable 1 - Statistical analysis comprised of linear mixed effects model regression (LMER) estimate, multiple comparison contrast tests between treatment groups, and analysis of variance utilizing type II sum of squares.

Sample of Unpleasant and Pleasant questions:

Pleasant questions:

1. What's one of your favorite memories from the last several years?
2. Could you describe your favorite hobbies?
3. Please tell me about one thing you are looking forward to in the upcoming year.
4. Where or what do you consider to be your happy place? Can you tell me about it?

Unpleasant questions:

1. Have you ever felt the need to conceal your blepharospasm from others, or are worried about how others would react to you? How did this make you feel?
2. Has it been difficult for you to do things that you normally enjoy due to your blepharospasm? If so, please describe a specific situation or leisure activity.
3. Have you avoided certain social situations where many people are present due to your symptoms? How often does this happen?
4. When do you feel your symptoms are at their worst? Can you describe those symptoms and what they're like?
